# Consumption

**Published:** 1878-05

**Authors:** 


The first foundations of consumption are laid in the want of free breathing; the consequence, instantaneous and inevitable is, that the little blood-vessels stretching along a distended air-cell become tortuous, winding, doubling, thus retarding the flow of blood, and *retardation is death.* The moment the life-blood stagnates, that moment it begins to die, and in approaching actual stagnation it becomes corrupt, impure, thick. But more blood coming in from behind, the pressure becomes greater, the sides of the blood-vessels become distended and at last begin to yield, and there is an oozing through of the more liquid portions of the blood in the shape of distinct atoms or drops, which as they ooze, become hard, as the gum does from a puncture of the bark of some tree; this oozed blood-particle, hardened, is the hateful TUBERCLE, the seed of consumption and death.

An atom ever so small takes up room, and millions of them amount to a great deal, hence the room, in the air-cells, already diminished by the want of full breathing, and further by the detention of the blood in the tortuous blood-channels, is still farther taken up by the hard tubercles, so that from the three causes, there is very little room for any air at all; thus it is that *shortness of breath* is never absent in any case of consumption. In fact, it is an early symptom, and comes on by slow degrees, so slow as to be imperceptible; *it comes on months before any cough is noticed.*

But another result springs from the increased diminution of room in the lungs, caused by tubercles of all sizes from a white mustard seed upwards; they help to intercept the flow of blood along the veins and arteries, and the pressure from behind still continuing, the blood-vessels cannot bear the strain, and burst, pouring out the blood into the lungs, that is, bleeding of the lungs, spitting of blood, *the forerunner of death.*

